# Copper pyrazole addition regulates soil mineral nitrogen turnover by mediating microbial traits

**DOI:** 10.3389/fmicb.2024.1433816

**Published:** 2024-10-01

**Authors:** Yuming Wang, Wenling Zhong, Xiwen Zhang, Minghui Cao, Zheng Ni, Mengxia Zhang, Jiangye Li, Yan Duan, Lifang Wu

**Affiliations:** ^1^The Centre for Ion Beam Bioengineering Green Agriculture, Hefei Institutes of Physical Science, Chinese Academy of Sciences, Hefei, China; ^2^Science Island Branch, Graduate School of USTC, Hefei, China; ^3^Zhongke Taihe Experimental Station, Taihe, China; ^4^School of Life Sciences, Anhui Agricultural University, Hefei, China; ^5^Institute of Agricultural Resources and Environment, Academy of Agricultural Sciences, Nanjing, China

**Keywords:** nitrification inhibitors, copper pyrazole, soil mineral nitrogen, microbial community, nitrogen-cycling genes

## Abstract

The huge amount of urea applied has necessitated best-developed practices to slow down the release of nitrogen (N) fertilizer while minimizing nitrate loss. However, the impact of nitrification inhibitors on mineral-N turnover and the associated microbial mechanisms at different stages remains unknown. A 60-day incubation experiment was conducted with four treatments: no fertilizer (CK), urea (U), urea with copper pyrazole (UC), and urea coated with copper pyrazole (SUC), to evaluate the changes about soil ammonia N ( ${NH}_{4}^{+}$ -N) and nitrate N ( ${\mathrm{NO}}_{3}^{-}$ -N) levels as well as in soil microbial community throughout the whole incubation period. The results showed that copper pyrazole exhibited significantly higher inhibition rates on urease compared to other metal-pyrazole coordination compounds. The soil ${NH}_{4}^{+}$ -N content peaked on the 10th day and was significantly greater in UC compared to U, while the ${\mathrm{NO}}_{3}^{-}$ -N content was significantly greater in U compared to UC on the 60th day. Copper pyrazole mainly decreased the expression of nitrifying (AOB-*amoA*) and denitrifying (*nirK*) genes, impacting the soil microbial community. Co-occurrence network suggested that *Mycobacterium* and *Cronobacter sakazakii-*driven Cluster 4 community potentially affected the nitrification process in the initial phase, converting ${NH}_{4}^{+}$ -N to ${\mathrm{NO}}_{3}^{-}$ -N. *Fusarium-*driven Cluster 3 community likely facilitated the denitrification of ${\mathrm{NO}}_{3}^{-}$ -N and caused N loss to the atmosphere in the late stage. The application of copper pyrazole may influence the process of nitrification and denitrification by regulating soil microbial traits (module community and functional genes). Our research indicates that the addition of copper pyrazole alters the community function driven by keystone taxa, altering mineral-N turnover and supporting the use of nitrification inhibitors in sustainable agriculture.

## 1 Introduction

Nitrogen (N) fertilizer plays a pivotal role in promoting soil productivity and maintaining soil health (Singh and Verma, 2007). Global N fertilizer consumption has exceeded 110 million tons per year and continues to increase (Hu et al., 2023). After they are applied to soil, N-components in fertilizers are transformed into the plant-available forms of ammonium-N ( ${NH}_{4}^{+}$ -N) and nitrate-N ( ${\mathrm{NO}}_{3}^{-}$ -N) through nitrification (Subbarao et al., 2006; Adhikari et al., 2021; Hosseini et al., 2023). However, frequent application of N fertilizer may decrease N utilization efficiency, cause soil acidification, and even reduce soil quality and functionality (Chien et al., 2016; Hu et al., 2023). Generally, only slightly over 35% of the urea applied is utilized under field conditions (Al-Juthery et al., 2021), and most of the N is lost in the form of ${\mathrm{NO}}_{3}^{-}$ , NH_3_, N_2_O, and N_2_, which leads to resource wastage (Al-Juthery et al., 2021; Mahmud et al., 2021; Upadhyay et al., 2023). Therefore, methods to improve N utilization efficiency is currently an urgent research issue worldwide.

Current strategies for reducing soil N loss and enhancing soil N fertilizer use efficiency mainly include the breeding of new plant varieties, optimizing N fertilizer regimes, diversifying crop rotations or intercropping, and using efficient fertilizers with urease and/or nitrification inhibitors (Wing et al., 2018; Woodward et al., 2021; Gao et al., 2022; Liu et al., 2022). Nitrification inhibitors show strong potential for improving N utilization efficiency and increasing crop yields, and the use of these agents are accepted by most farmers (Mahmud et al., 2021; Liu Y. et al., 2014). To the best of our knowledge, pyrazole and its derivatives, as one type of nitrification inhibitors, have a potent inhibitory effect on nitrification (Mccarty et al., 1989; Woodward et al., 2021; Jog et al., 2022; Li et al., 2022), which have been widely used in Europe (Gilsanz et al., 2016) and have since been widely used for yield improvement (Woodward et al., 2021). The mechanisms underlying nitrification inhibitor function primarily include direct suppression of nitrification via ammonia monooxygenase (AMO) deactivation, in addition to shifts in the soil microbial community structure (Adhikari et al., 2021; Woodward et al., 2021). Microorganisms, as the main drivers of soil elemental cycling, have received increasing amounts of attention due to their ability to mediate N turnover and have become a hotspot of current research (Adhikari et al., 2021; Gupta et al., 2023). Hence, deciphering the relationship between soil microbial functions and N turnover is particularly crucial for ensuring soil health.

Many authoritative studies suggest that soil microorganisms are involved in the soil N cycle via processes such as biological N fixation, ammonification, nitrification, and denitrification (Liu W. et al., 2014; Woodward et al., 2021). Generally, when urea is applied to soil, a small amount of the ${NH}_{4}^{+}$ -N formed can be absorbed and utilized by plants, and most of the ${NH}_{4}^{+}$ -N is converted into ${\mathrm{NO}}_{3}^{-}$ -N by ammonia oxidizer (ammonia oxidizer archaea [AOA] and ammonia oxidizer bacteria [AOB])-driven nitrification (Saud et al., 2022). Subsequently, most ${\mathrm{NO}}_{3}^{-}$ -N is released as NO_2_ and N_2_ from the soil due to denitrifying bacteria-driven denitrification (Wrage et al., 2001). Moreover, nitrification inhibitors slow the initial step of nitrification by reducing the abundance and activity of AOB so that more ${NH}_{4}^{+}$ -N can be taken up and utilized by plant roots (Topp and Knowles, 1984). In addition, specific microbial species have been indicated to be involved in soil N turnover (Cáceres et al., 2018; Woodward et al., 2021). For example, Li et al. (2018) showed that *Stenotrophomonas*, a typical denitrifier, was the keystone taxa contributing to N_2_O production. Additionally, Van Kessel et al. (2015) reported the enrichment and initial characterization of two *Nitrospira* species that encode AMO necessary for ammonia oxidation via nitrite to nitrate in their genomes. Related studies have shown that nitrification inhibitors can limit the activity of such functional microbes, altering N transformation and enhancing fertilizer use efficiency. Therefore, elucidating the effect of nitrification inhibitor application on microbial functions during different periods is critical.

On the other hand, soil urease activity is a crucial factor influencing N turnover. Urease catalyzes the hydrolysis of urea into NH_3_, thereby reducing the efficiency of urea utilization by plants (Li et al., 2019). Therefore, decreasing urease activity is an important strategy for (further) improving urea utilization efficiency. Research has shown that urease is sensitive to heavy metal ions, which can inhibit urease activity (Hemida et al., 1997; Yang et al., 2006), slowing down the decomposition of N fertilizers and enhancing their utilization efficiency.

Furthermore, some studies have suggested that coated slow-release materials (Naz and Sulaiman, 2016) may be suitable options for nitrification inhibitors. In addition, slow-release material could release urea continuously to ensure the effectiveness of the fertilizer (Naz and Sulaiman, 2016). However, the effects of nitrification inhibitors chelated with heavy metal on soil mineral N content and microbial traits during different periods are poorly understood. Therefore, we conducted a 60-day incubation experiment to (1) determine the dynamics of mineral N content and microbial traits (including N-cycling gene abundance and community) after nitrification inhibitor application and (2) clarify the linkages between mineral N content and microbial traits at different periods. We hypothesized that (i) soil ${NH}_{4}^{+}$ -N and ${\mathrm{NO}}_{3}^{-}$ -N contents as well as microbial N-cycling gene abundance would show distinct responses to different treatments during incubation and (ii) soil ${NH}_{4}^{+}$ -N and ${\mathrm{NO}}_{3}^{-}$ -N turnover were closely related to specific microbial taxa.

## 2 Materials and methods

### 2.1 Experimental soil and material preparation

The test soil was obtained from the topsoil (0–20 cm) of a wheat field in Shushan District, Hefei city, Anhui Province, China (31° 90 W′, 117° 17 E′). The soil samples were air-dried, ground, passed through a sieve of 2 mm and stored at room temperature. The soil chemical properties were as follows: 6.3 pH (W/V, 1:2.5), 7.39 g·kg^−1^ organic carbon, 0.39 g·kg^−1^ total phosphorus, and 1.05 g·kg^−1^ total N.

The metal-pyrazole coordination compounds were prepared using the solvent evaporation method (Das et al., 2010; Zhai, 2020; Li et al., 2022), with CuCl·2H_2_O, ZnCl_2_, CoCl_2_·6H_2_O, and CdI_2_ as raw materials to form metal-pyrazole coordination compounds. The yield of all metal-pyrazole coordination compounds exceeded 60%, and the specific method can be found in Supplementary Text S1.

Urease activity was assessed using the spectrophotometric method (Fahey et al., 2013). Take 25 μL of urease solution (10 KU/L) in a centrifuge tube and add 25 μL of metal-pyrazole coordination compounds of different concentrations. Place the tubes in a 25°C incubator for 0.5 h. Then add 200 μL of a buffer solution containing urea and phenol red indicator, mix well, and incubate in a 25°C incubator for another 0.5 h. Measure the absorbance of the samples at a wavelength of 570 nm using a spectrophotometer, and calculate the urease inhibition rate using the following formula:


(1) $$\mathrm{Urease inhibition rate}\;\left(\%\right)=\frac{\left(A-B\right)}{A}\times 100\%$$

Where: A is the difference in absorbance before and after the reaction in the sample solution without added urease inhibitor, and B is the difference in absorbance before and after the reaction in the sample solution with added urease inhibitor.

Synthesis of urea coated with copper pyrazole slow-release fertilizer: The speed of the sugar-coating machine (BY-300, Taizhou Jincheng Pharmaceutical Machinery Co., Ltd., China) was adjusted to 45 r/min, the blower was turned on, 250 g of urea was added to the sugar-coating machine, and it was heated at 70°C for 3 min. Then, the heating and blowing were stopped, 11.70 g of copper pyrazole was added to the sugar-coating machine and evenly mixed, and a small amount of NaOH solution (pH = 9.5) was sprayed several times until the urea particles did not stick. Next, 112 g of potato starch was added to the sugar-coating machine and mixed evenly, 50 mL of epichlorohydrin and 50 mL of NaOH solution (pH = 9.5) were sprayed into the sugar-coating machine several times, and the urea coated with copper pyrazole slow-release fertilizer was obtained after drying with a blower for 30 min. All the above chemical reagents (analytical purity) were purchased from Sinopharm Chemical Reagents Co., Ltd. Shenggong Bioengineering Co., Ltd. (China).

### 2.2 Experimental design and soil mineral N analysis

Soil N transformation and microbial trait changes were assessed via a destructive incubation experiment. Four treatments were used, namely, control (CK), urea (U), urea and copper pyrazole (UC), and urea coated with copper pyrazole slow-release fertilizer (SUC). Each experiment was conducted in triplicate to ensure the reliability of the results. Three hundred grams of soil was taken from each treatment and placed in a plastic box, and the corresponding materials (386.1 mg of urea, 386.1 mg of urea, 18 mg of copper pyrazole, and 483 mg of urea coated with copper pyrazole slow-release fertilizer) were added to each treatment and mixed homogeneously, followed by 15 parallel samples from each treatment. The mixture was incubated at 25°C for 60 days, after which the soil moisture was adjusted to 60% of the maximum water holding capacity every 7 days during incubation. Thirty grams of the soil from each treatment was collected on Days 0, 4, 10, 30, and 60. One part was kept at −80°C for microbial sequencing, and the other was used for the measurement of soil ${NH}_{4}^{+}$ -N and ${\mathrm{NO}}_{3}^{-}$ -N contents, which were determined by a Kelvin Nitrogen Determination Instrument (K-360, BUCHI BUCHI Laboratory Equipigs Co. Trading Ltd., Switzerland).

### 2.3 Quantitative PCR

Soil samples at the 10th and 60th days of incubation were selected for microbial analysis, DNA extraction of soil samples was done by Bemac Biotechnology Co Ltd. (China). The *amoA* gene encoding AMO and the *nirK* gene encoding nitrite reductase and the *nosZ* gene encoding nitrous oxide reductase were analyzed by a real-time fluorescence Quantitative PCR (qPCR) instrument (LightCycler^®^96, Roche, Switzerland). qPCR used three different primers - *bamoA_1F*/bamoA_2R (AOA-*amoA* and AOB-*amoA*), Cunir3F/Cunir3R (*nirK*) and *nosZ*-2F/*nosZ*-2R (*nosZ*)—to determine the copy numbers of the AOA-*amoA*, AOB-*amoA*, *nirK* and *nosZ* genes in this study (Chen Z. et al., 2021). The 20 μL qPCR mixtures contained 10 μL TB Green Premix Ex Taq polymerase (TaKaRa Bio, China), 0.5 μL each primer, 1 μL template DNA, and 8.5 μL nuclease-free water. Soil DNA was diluted prior to PCR to avoid co-extracted compound inhibition. External plasmid DNA standard curves with 10-fold serial dilutions were utilized to quantify gene copies. The qPCR conditions was initiated at 95°C for 10 min; 40 cycles of 95°C for 30 s, 55°C for 30 s and 68°C for 40 s of extension (AOA-*amoA*, AOB-*amoA*); 40 cycles of 95°C for 60 s, 57°C for 30 s and 72°C for 60 s of extension (*nirK*); 40 cycles of 95°C for 60 s, 56°C for 30 s and 72°C for 60 s of extension (*nosZ*). Melt curve analysis confirmed PCR specificity. qPCR was performed in triplicate, and amplification efficiencies of 95–105% were obtained with *r*^2^ values > 0.99 for all microbial functional genes.

### 2.4 Amplicon sequencing of the bacterial 16S rRNA and fungal ITS rRNA genes

High-throughput sequencing was performed with the Illumina MiSeq sequencing platform (Illumina Inc.). The primers 341F/806R (5′-CCTAYGGGRBGCASCAG-3′/5′-GGACTACNNGGGTATCTAAT-3′) were chosen to amplify the 16S rRNA genes in the V3–V4 hypervariable region (Chen K. H. et al., 2021). A unique 5-bp barcode sequence was added to the forward primers to distinguish the PCR products from different samples. PCR was conducted in a 50-μL reaction mixture containing 27 μL of ddH_2_O, 2 μL (5 μM) of each forward/reverse primer, 2.5 μL (10 ng) of template DNA, 5 μL (2.5 mM) of deoxynucleoside triphosphates, 10 μL of 5× Fastpfu buffer, 0.5 μL of bovine serum albumin, and 1 μL of TransStart Fastpfu polymerase (TransGen, Beijing, China). The PCR conditions were 94°C for 5 min; 30 cycles of 94°C for 30 s, 52°C for 30 s and 72°C for 30 s of extension; followed by 72°C for 10 min. The reaction products were pooled and purified using the QIAquick PCR urification kit (Qiagen), and they were quantified using a NanoDrop ND-1000 (Thermo Scientific). After the individual quantification step, amplicons were pooled in equal amounts, and paired-end 2,300-bp sequencing was performed using the Illumina MiSeq platform with MiSeq Reagent Kit v3. PCR products from all samples were pooled and purified in equimolar concentrations, and sequencing was performed on an Illumina MiSeq instrument.

The fungal ITS1 region was amplified via polymerase chain reaction (PCR) using the ITS1F (CTTGGTCATTTAGAGGAAGTAA) and ITS2 (GCTGCGTTCTTCATCGATGC) primers (Ghannoum et al., 2010; Duan et al., 2023). The 50 μL PCR reaction mixture consisted of 1 μL (30 ng) template DNA, 4 μL (1 μM) of each forward and reverse primer, 25 μL PCR Master Mix, and 16 μL ddH2O. The PCR conditions were 95°C for 5 min; 25 cycles of 94°C for 30 s, 50°C for 30 s and 72°C for 40 s of extension; followed by 72°C for 7 min. PCR products from all samples were pooled and purified in equimolar concentrations, and sequencing was performed on an Illumina MiSeq instrument (Illumina, San Diego, CA, United States). The raw sequence data were processed using the Qualitative Insights into Microbial Ecology (QIIME) pipeline. Poor-quality sequences with lengths less than 200 bp and quality scores less than 20 were discarded, and the chimeras were removed using the UCHIME algorithm (Edgar, 2010). The remaining sequences were assigned to operational taxonomic units (OTUs) with a 97% similarity threshold using UCLUST. Taxonomy was assigned using the UCLUST consensus taxonomic assigner algorithm and the UNITE fungal database (Abarenkov et al., 2010).

### 2.5 Statistical analysis and network construction

Before statistical analysis, the normality and homogeneity of variance were tested by the Kolmogorov–Smirnov test and Levene’s test, respectively. If normality was not met, log or square-root transformation was performed. Analysis of variance (ANOVA) was used via Duncan’s test at *p* < 0.05 in IBM SPSS v26.0 to compare significant differences in ${NH}_{4}^{+}$ -N and ${\mathrm{NO}}_{3}^{-}$ -N content and AOA-*amoA*, AOB-*amoA*, *nirK* and *nosZ* gene abundance among the treatments. ANOVA was performed in SPSS ver. 27.0 (SPSS, Inc., Chicago, IL, United States).

Simpson’s index was calculated to represent the alpha diversity of soil bacteria and fungi via the R package “vegan.” Changes in the community structure of the soil bacteria and fungi were evaluated using principal coordinate analysis (PCoA), and Adonis analysis was used to test for significant differences in the bacterial and fungal community structures between the treatments. A chord chart was generated by Wekemo Bioincloud^1^ to visualize the main phyla and genera of bacteria and fungi. Variation in bacterial and fungal communities was quantified using PCoA Axis 1, and linear regression was used in Origin 2021 to establish associations between soil ${NH}_{4}^{+}$ -N and ${\mathrm{NO}}_{3}^{-}$ -N content and between different network cluster community variations and determine the relationships between specific network clusters and soil mineral N content.

To characterize the patterns of soil microbial interactions during the N cycle, we constructed a co-occurrence network with the R packages “igraph” and “WGCNA.” We constructed microbial networks using bacteria and fungi with relative abundances greater than 0.01%, screened nodes with Pearson’s correlation coefficients greater than 0.6 and *p* < 0.05, performed modular analysis based on the connectivity between nodes, visualized the network through Gephi (ver. 0.10), and calculated information on network topological features (Ma et al., 2021). The within-cluster connectivity (*Zi*) and among-cluster connectivity (*Pi*) of different clusters were calculated using MENA^2^ (Ma et al., 2021) and filtered for peripherals (*Zi* ≤ 2.5, *Pi* ≤ 0.62), connectors (*Zi* ≤ 2.5, *Pi* > 0.62), cluster hubs (*Zi* > 2.5, *Pi* ≤ 0.62) and network hubs (*Zi* > 2.5, *Pi* > 0.62) (Deng et al., 2012).

Random forest (RF) analyses were performed to predict the relative importance of keystone taxa to soil ${NH}_{4}^{+}$ -N and ${\mathrm{NO}}_{3}^{-}$ -N content via the R package “randomForest.” Heatmaps were constructed to reveal the relationships between the relative abundance of ${NH}_{4}^{+}$ -N and ${\mathrm{NO}}_{3}^{-}$ -N in Origin 2021. The enrichment of keystone taxa in the different treatment groups was visualized using bubble plots in Sangerbox^3^ (Shen et al., 2022).

## 3 Results

### 3.1 Synthesis and selection of metal pyrazole coordination compounds

Metal pyrazole coordination compounds were synthesized using cobalt, copper, zinc, and cadmium with pyrazole (Supplementary Figure S1), and their urease inhibition rates were evaluated at a concentration of 100 μmol/L. The results indicated that the copper pyrazole exhibited the most potent inhibitory effect on urease, significantly surpassing the other compounds (*p* < 0.05), with inhibition rates ranging from 9.30 to 22.68 times higher than those of the other groups (Supplementary Table S1, Equation 1). Subsequently, we determined the half-maximal inhibitory concentration (IC50) of the copper pyrazole compound on urease, using acetohydroxamic acid as a positive control. The findings (Supplementary Figure S2) revealed that the IC50 of the pyrazole-copper complex was 0.27 μmol/L, markedly lower than the 6.30 μmol/L of the positive control (*p* < 0.001), representing only 4.29% of the positive control value. Consequently, we selected the pyrazole-copper complex for further research and analysis.

### 3.2 Soil mineral N turnover during incubation

**Figure 1:**
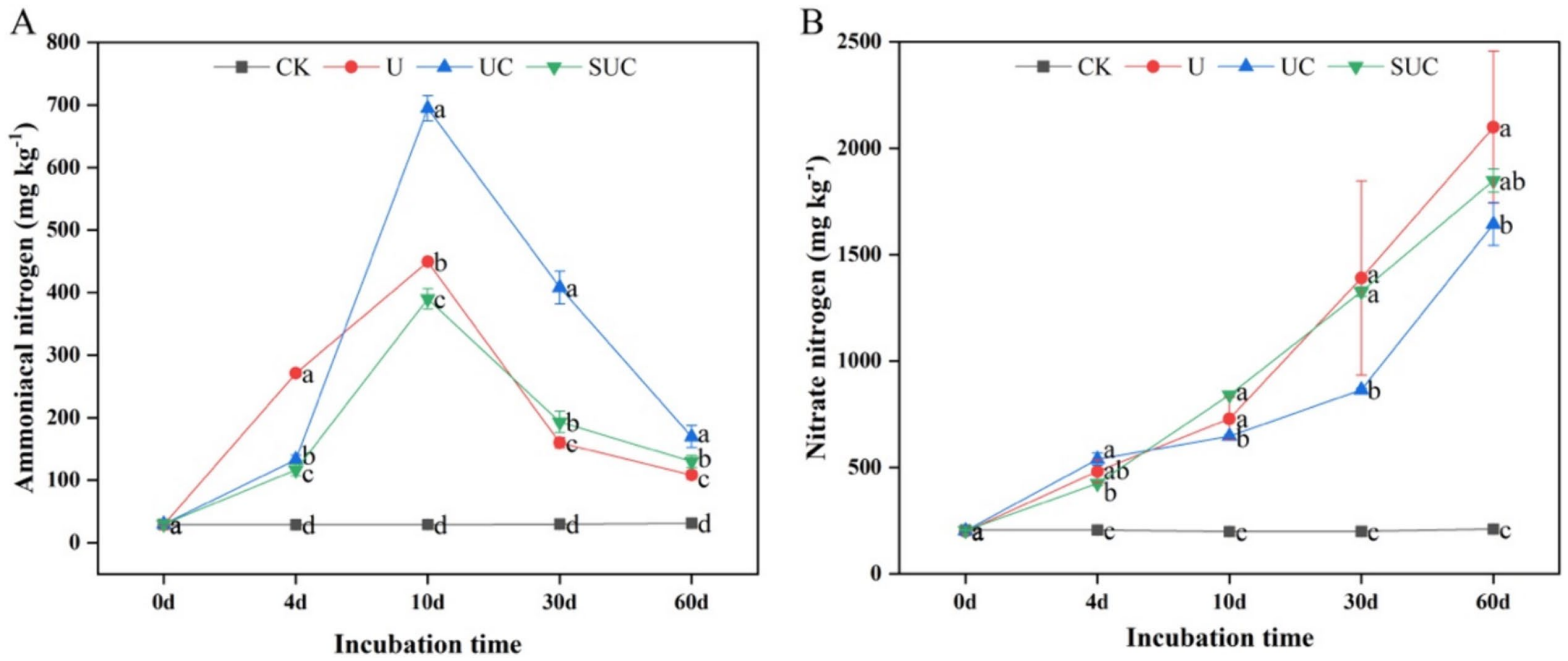
Dynamics of the soil ${NH}_{4}^{+}$ -N and ${\mathrm{NO}}_{3}^{-}$ -N contents in the different treatment groups on the 10th day **(A)** and the 60th day **(B)**. Different letters represent significant differences at *p* < 0.05 under different treatments at the same incubation time. CK, control; U, urea; UC, urea with copper pyrithioxin; SUC, urea coated with copper pyrithioxin.

Nitrification inhibitors had notable effects on the soil mineral N content during incubation. The UC and SUC treatments changed the contents of ${NH}_{4}^{+}$ -N and ${\mathrm{NO}}_{3}^{-}$ -N during the incubation period (Figure 1). Compared with those in the CK treatment, the contents of ${NH}_{4}^{+}$ -N and ${\mathrm{NO}}_{3}^{-}$ -N in each treatment were significantly greater (*p* < 0.05). The ${NH}_{4}^{+}$ -N content increased with incubation time, peaked on the 10th day after treatment, and then declined until the end of the incubation. On the 10th day, the UC treatment had the highest ${NH}_{4}^{+}$ -N content, which was significantly greater than that in the other treatments (*p* < 0.05) followed by U, SUC and CK; on the 60th day, the ${\mathrm{NO}}_{3}^{-}$ -N content in each treatment (except for CK) increased gradually with time, and the maximum value was shown on the 60th day. The ${\mathrm{NO}}_{3}^{-}$ -N content in the U treatment was significantly greater than that in the other treatments (*p* < 0.05), followed by that in the SUC, UC and CK treatments.

### 3.3 Soil N-cycling gene abundance and microbial community

The soil microbial traits exhibited different responses to each treatment (Table 1). The UC and SUC treatments significantly decreased nitrification and denitrification gene abundance (*p* < 0.05). On the 10th day, compared to those in the U treatment, the UC and SUC treatments significantly decreased the AOA-*amoA* gene abundance by 53.80 and 16.85%, respectively (*p* < 0.05); UC and SUC significantly decreased the AOB-*amoA* gene abundance by 95.91 and 92.05%, respectively (*p* < 0.05). The UC treatment decreased the *nosZ* gene abundance by 57.66%, which was significantly lower than that in the U treatment (*p* < 0.05). Similarly, on the 60th day, the *nirK* gene abundance was significantly lower (by 19.36 and 54.32%) in the UC (*p* > 0.05) and SUC (*p* < 0.05) groups, respectively, than in the U treatment group. Additionally, on the 60th day, the abundance of *nosZ* in the UC and SUC treatments was significantly higher than (that) in the U and CK treatments (*p* < 0.05), which is the opposite of what was observed on the 10th day.

**Table 1:** Abundances of soil N-cycling genes under different treatments during incubation.

Cultivation time	Treatments	AOA-*amoA* (×10^5^ copies g^−1^ dry soil)	AOB-*amoA* (×10^5^ copies g^−1^ dry soil)	*nirK* (×10^5^ copies g^−1^ dry soil)	*nosZ* (×10^5^ copies g^−1^ dry soil)
10th day	CK	9.34 ± 2.05 a	4.59 ± 1.22 b	53.57 ± 10.60 ab	9.52 ± 1.47 a
	U	7.36 ± 0.60 ab	39.63 ± 4.08 a	28.51 ± 3.24 c	10.70 ± 0.61 a
	UC	3.40 ± 1.28 c	1.62 ± 0.66 b	46.97 ± 1.49 b	4.53 ± 1.14 b
	SUC	6.12 ± 1.10 b	3.15 ± 0.15 b	62.84 ± 4.75 a	10.55 ± 0.31 a
60th day	CK	1.59 ± 0.04 c	1.53 ± 0.35 c	68.23 ± 8.25 a	3.89 ± 0.95 c
	U	19.52 ± 2.27 b	132.73 ± 20.62 a	67.98 ± 8.83 a	3.94 ± 0.12 c
	UC	34.99 ± 1.91 a	85.45 ± 4.73 b	54.82 ± 2.82 a	6.25 ± 0.13 b
	SUC	3.75 ± 0.41 c	1.89 ± 1.25 c	31.05 ± 5.77 b	9.38 ± 0.34 a

Values are means ± standard deviation (*n* = 3) and different letters within the same column denote significant differences (*p* < 0.05) under different treatments. CK, Control; U, urea; UC, urea with copper pyrithioxin; SUC, urea coated with copper pyrithioxin.

**Figure 2:**
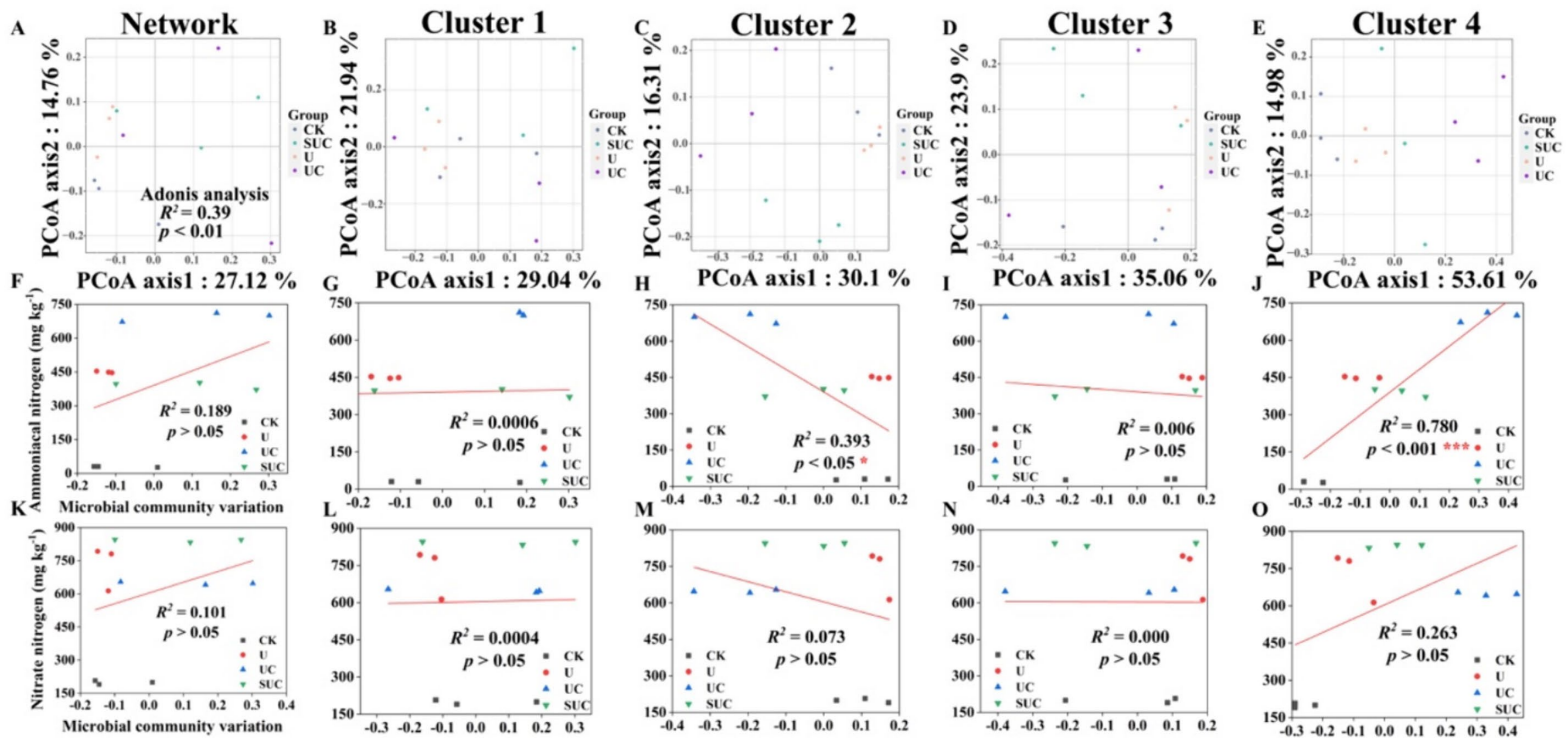
PCoA of the community structures of soil microorganisms and linear regression analysis of microbial community variation and ${NH}_{4}^{+}$ -N and ${\mathrm{NO}}_{3}^{-}$ -N contents after the 10th day of cultivation. PCoA analyses of the whole microbial community and the Cluster 1 to Cluster 4 community **(A–E)**; linear regression analyses of the microbial community and ${NH}_{4}^{+}$ -N content at the cluster scale **(F–J)**; and linear regression analyses of microbial community change and ${\mathrm{NO}}_{3}^{-}$ -N content at the cluster scale **(K–O)**. CK, control; U, urea; UC, urea with copper pyrithioxin; SUC, urea coated with copper pyrithioxin.

**Figure 3:**
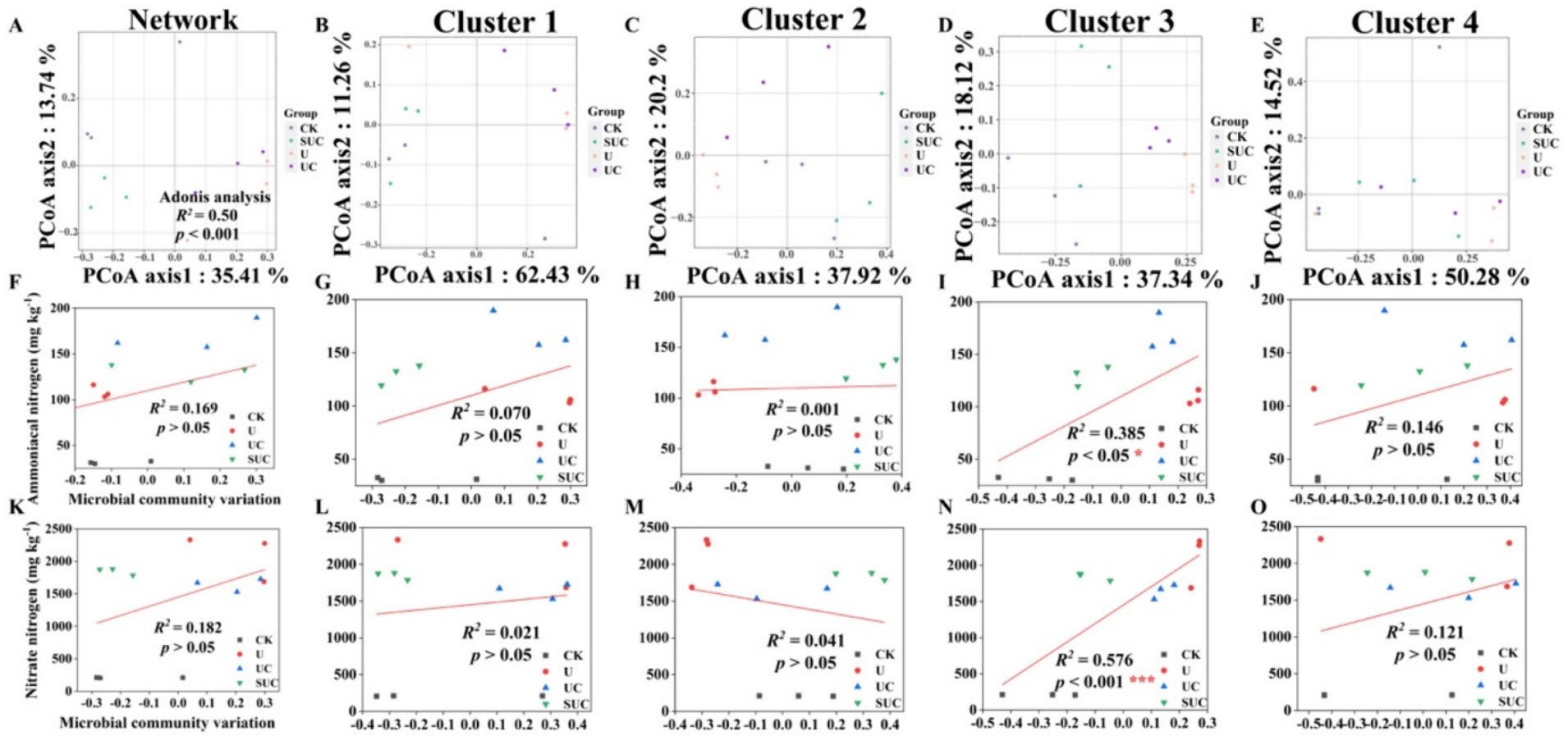
PCoA of the community structures of soil microorganisms and linear regression analysis of microbial community variation and ${NH}_{4}^{+}$ -N and ${\mathrm{NO}}_{3}^{-}$ -N contents after the 60th day of cultivation. PCoA analyses of the whole microbial community and the Cluster 1 to Cluster 4 community **(A–E)**; linear regression analyses of the microbial community and ${NH}_{4}^{+}$ -N content at the cluster scale **(F–J)**; and linear regression analyses of microbial community change and ${\mathrm{NO}}_{3}^{-}$ -N content at the cluster scale **(K–O)**. CK, control; U, urea; UC, urea with copper pyrithioxin; SUC, urea coated with copper pyrithioxin.

Soil microbial diversity and community composition varied among the different treatments (Figures 2A, 3A; Supplementary Table S2). For bacteria, the *α*-diversities (Simpson’s indices) of the UC and SUC treatments were significantly lower than those of the CK and U treatments on the 10th day (*p* < 0.05), whereas there was no significant difference on the 60th day. There was no significant difference in the fungal Simpson’s indices under the different treatments at the 10th and 60th days.

The changes in soil microbial community composition were also visualized by PCoA (Figures 2A, 3A), and Adonis analysis also revealed significant differences in the soil microbial community among the treatments on the 10th and 60th days (*R*^2^ = 0.39, *p* < 0.01; *R*^2^ = 0.50, *p* < 0.001; Figures 2A, 3A).

### 3.4 Microbial co-occurrence network

**Figure 4:**
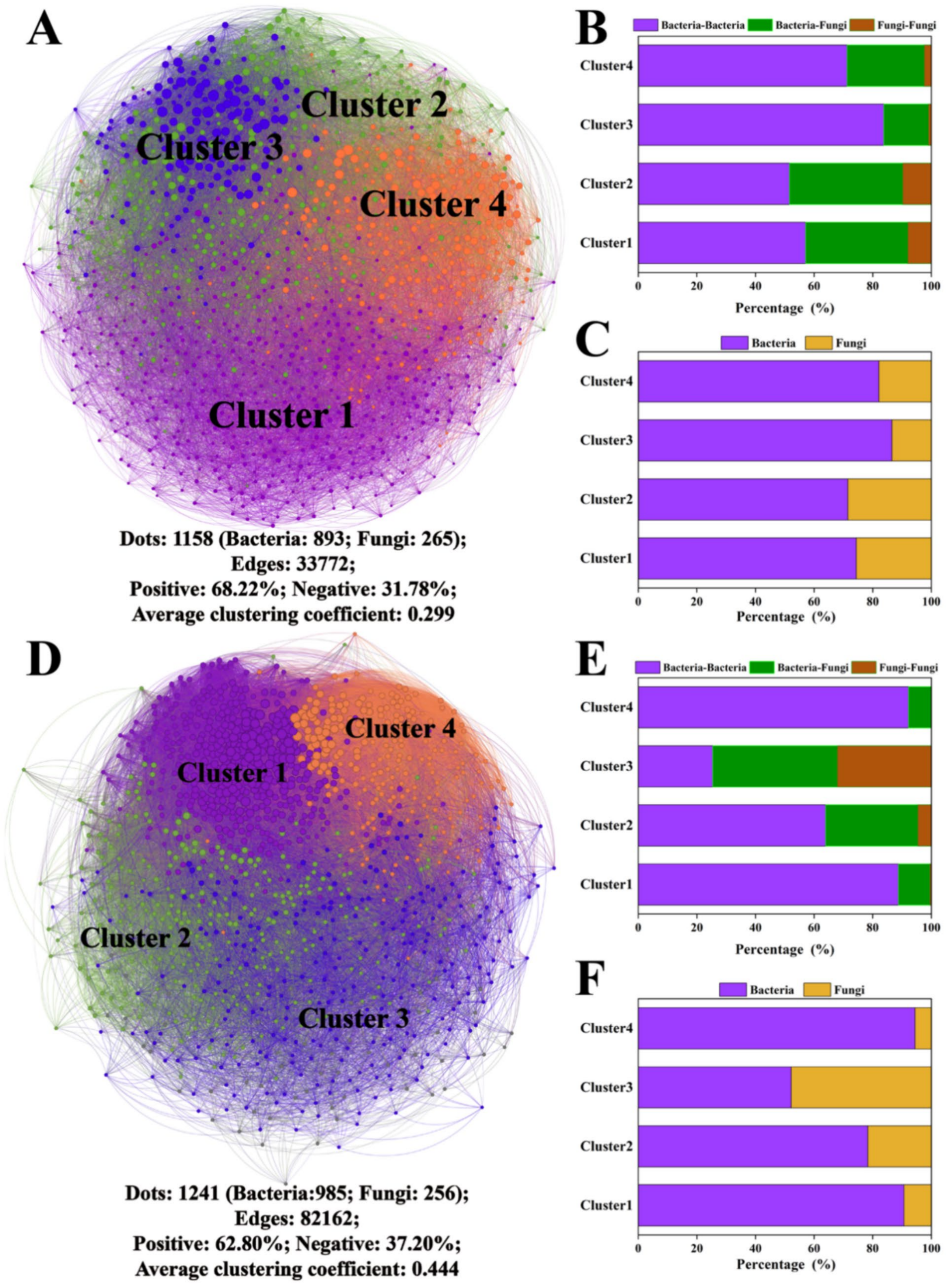
Microbial network analysis after the 10th and 60th days of cultivation. Microbial networks on the 10th day **(A)** and 60th day **(D)**; the proportions of connections between different bacterial and fungal ASVs within the network on the 10th day **(B)** and 60th day **(E)**; and the proportions of bacterial and fungal ASVs within the network on the 10th day **(C)** and 60th day **(F)**. The different colors of the nodes in the microbial networks represent different clusters.

A series of co-occurrence networks were constructed on the 10th and 60th days, and the topological characteristics of the network are shown in Figure 4. The microbial network on the 10th day was divided into four main clusters, and the bacterial population was larger than the fungal population. The correlation between ASVs was mainly positive (68.22%), with an average network clustering coefficient of 0.229. On the 60th day, there were four dominant clusters in the microbial network containing predominantly positive correlations (62.80%), with an average network clustering coefficient of 0.444. Overall, the percentage of edges linking bacteria and fungi in the network was 7.41–28.74% greater than that for fungus-to-fungus connections. The specific network cluster topology features of the above clusters are shown in Supplementary Figure S3.

**Figure 5:**
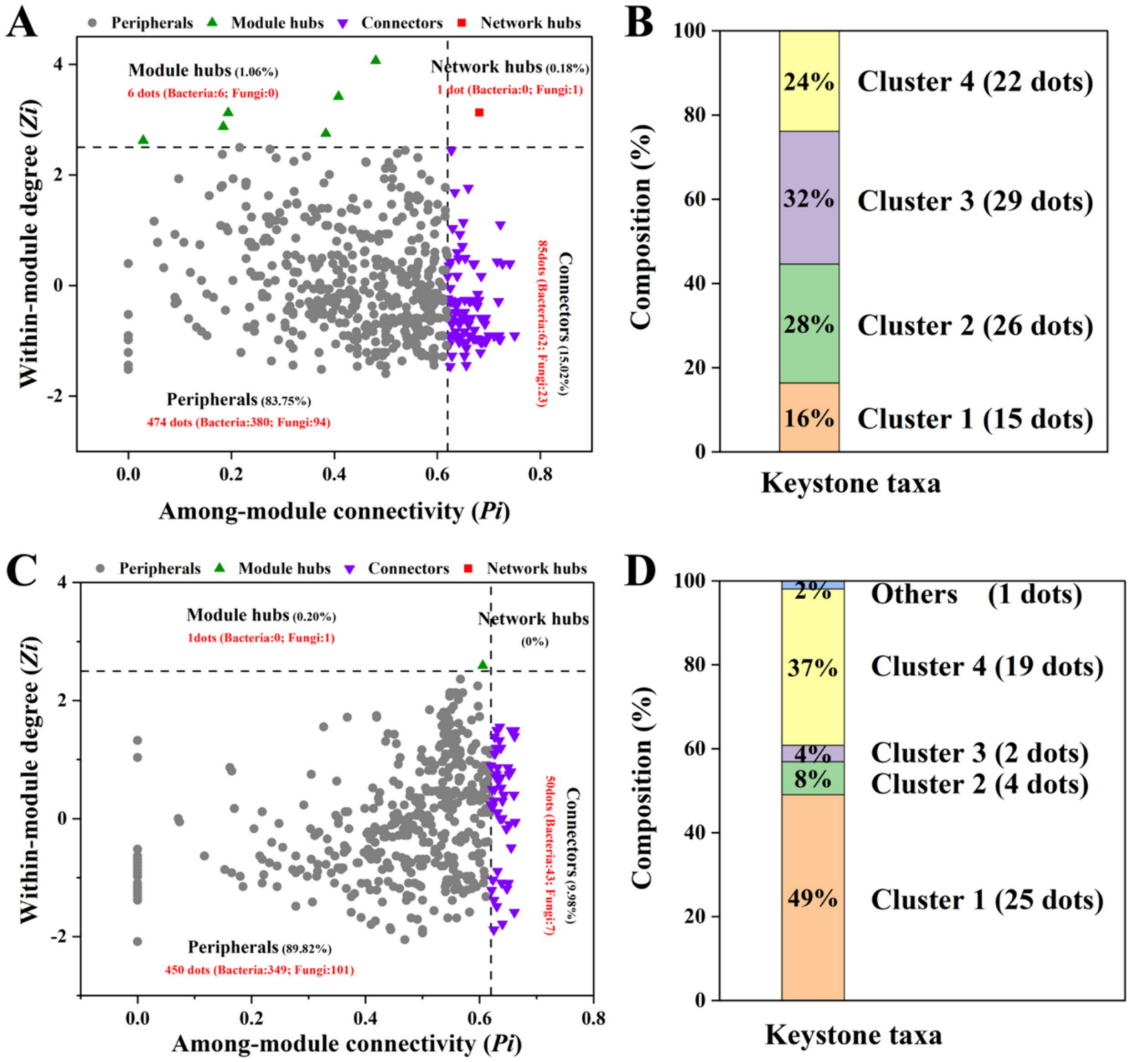
*Zi–Pi* plot showing the keystone taxa in the different microbial networks on the 10th day **(A)** and 60th day **(C)**. The proportion of keystone taxa in different network clusters on the 10th day **(B)** and the 60th day **(D)** of incubation.

*ZP* plots were constructed to identify the topological roles of each node in the network (Figure 5). On the 10th day, 92 species were detected as keystone taxa. There were 1, 6 and 85 taxa observed within network hubs, cluster hubs and connectors, respectively (Figure 5A). The distribution of microbial keystone taxa was 16% in Cluster 1, 28% in Cluster 2, 29% in Cluster 3 and 24% in Cluster 4 (Figure 5B). These bacteria belonged to the bacterial phylum Proteobacteria (49%) and the fungal phylum Ascomycota (71%) (Supplementary Table S3). On the 60th day, there were 51 keystone taxa in all the clusters, and 0, 1 and 50 taxa were observed in the network hubs, cluster hubs and connectors, respectively. The distribution of microbial keystone taxa was 49% in Cluster 1 and 37% in Cluster 4 (Figure 5C). The keystone taxa mainly belonged to the bacterial phylum Proteobacteria (58%) and the fungal phylum Ascomycota (50%) (Supplementary Table S3). The taxonomic information of soil keystone taxa is shown in Supplementary Table S4.

### 3.5 Relationships between soil microbial traits and mineral N

Soil microbial N-cycling genes may participate in mineral N turnover. A heatmap was generated (Supplementary Figure S4), which shows that the AOA-*amoA* gene abundance was negatively correlated with the ${NH}_{4}^{+}$ N content (*p* < 0.001) on the 10th day, while the AOA-*amoA* gene abundance was positively correlated with the ${NH}_{4}^{+}$ N content (*p* < 0.01) on the 60th day. The *NirK* gene abundance was negatively correlated with the ${NH}_{4}^{+}$ -N content on the 60th day (*p* < 0.05).

Linear regression (Figures 2, 3) revealed that the soil ${NH}_{4}^{+}$ -N content was significantly correlated with the microbial community variation in Clusters 2 and 4 on the 10th day (*R*^2^ = 0.393, *p* < 0.05 for Cluster 2; *R*^2^ = 0.780, *p* < 0.001 for Cluster 4). On the 60th day, the soil ${NH}_{4}^{+}$ -N and ${\mathrm{NO}}_{3}^{-}$ -N contents were significantly positively correlated with those in Cluster 3 (*R*^2^ = 0.385, *p* < 0.05 for ${NH}_{4}^{+}$ -N content; *R*^2^ = 0.576, *p* < 0.001 for ${\mathrm{NO}}_{3}^{-}$ -N content).

It is well known that keystone taxa are crucial drivers of specific cluster communities and affect the function of the whole network. Therefore, it was necessary to clarify the effect of keystone taxa on mineral N turnover during incubation. A series of random forest models (Supplementary Figure S5) were used to determine the relative importance of the selected keystone taxa for the soil mineral N content. We selected 5 bacterial (BASV159, BASV661, BASV129, BASV430, and BASV197) and fungal (FASV59 FASV39, FASV65, FASV69, and FASV4) keystone taxa with the highest relative abundance within Cluster 2 and 4 on the 10th day for further study. A heatmap (Supplementary Figure S6) showed that the relative abundances of BASV159, BASV661 BASV129, BASV430, FASV59 and FASV39 were significantly negatively correlated with the ${NH}_{4}^{+}$ -N content on the 10th day. Moreover, the relative abundance of FASV188 was significantly negatively correlated with the ${NH}_{4}^{+}$ -N content, while the relative abundances of FASV188 and FASV188 were significantly negatively correlated with the ${\mathrm{NO}}_{3}^{-}$ -N content on the 60th day.

**Figure 6:**
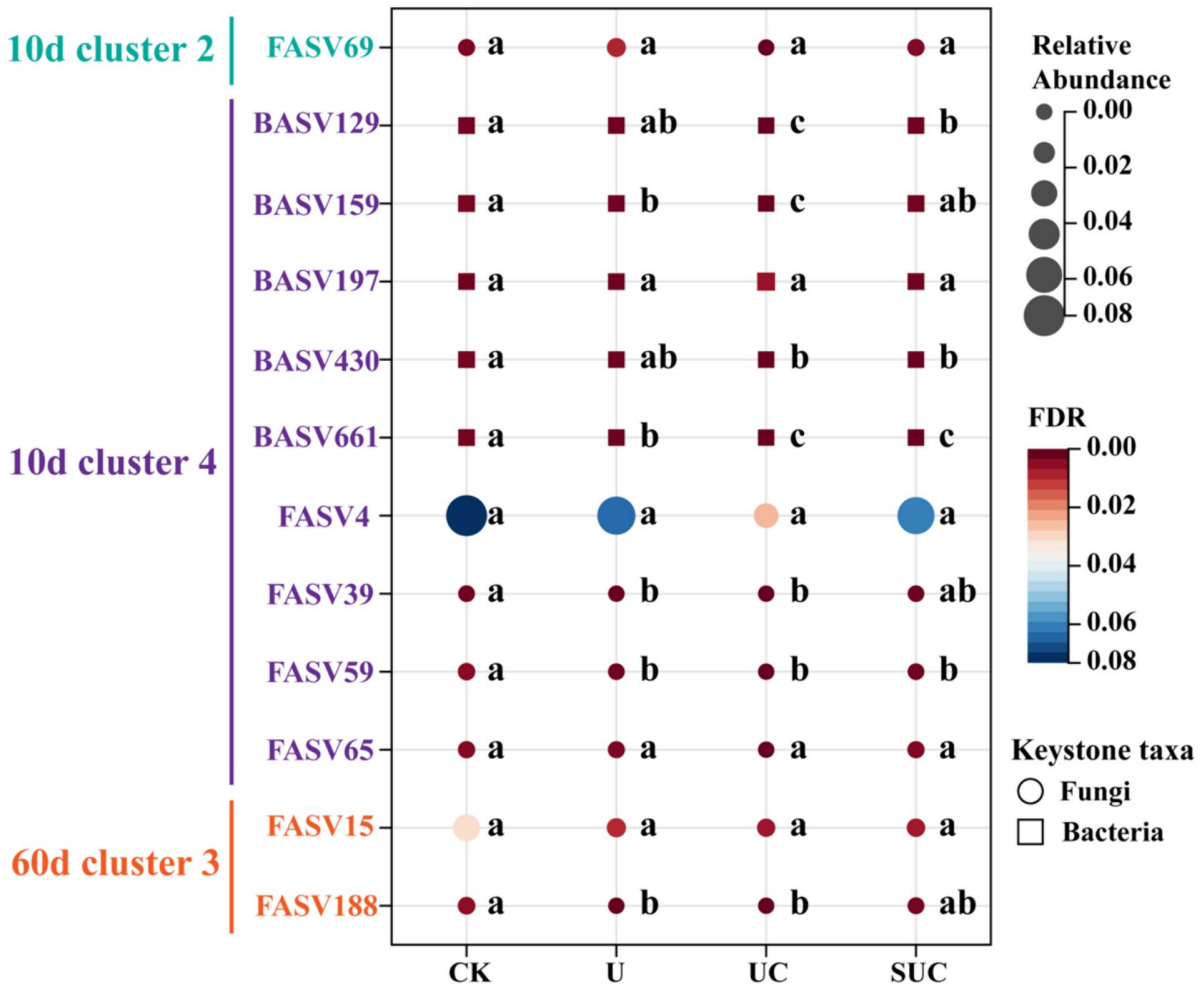
Bubble plot of the relative abundance of microbial keystone taxa under different treatments. The different colors and node sizes represent the relative abundance of keystone taxa in each treatment, with circles representing fungal taxa and squares representing bacterial taxa. Different letters in the same row denote significant differences (*p* < 0.05) among the different treatments. CK, control; U, urea; UC, urea with copper pyrithioxin; SUC, urea coated with copper pyrithioxin.

Furthermore, to visualize the changes in the relative abundance of the selected keystone taxa across treatments, a bubble plot was constructed. Figure 6 shows that, except for FASV69 and BASV197, the relative abundances of the keystone taxa in the CK treatment were greater than those in the other treatments. The relative abundances of BASV129, BASV159, BASV430, and BASV661 in the UC treatment were significantly lower (*p* < 0.05) than those in the U treatment.

## 4 Discussion

Generally, N leaching or mineralization in agroecosystems results in low N utilization efficiency (Al-Juthery et al., 2021). Nitrification inhibitor application can mediate the soil microbial community and function and thus has become an accepted way to address this issue (Tindaon et al., 2012; Adhikari et al., 2021; Mahmud et al., 2021; Woodward et al., 2021). Additionally, metal ions can inhibit urease activity, thereby slowing down the hydrolysis of urea (Li et al., 2019). The effects of different metal ions on urease are various; Zaborska et al. (2004) found that Cu^2+^ and Zn^2+^ exhibit the strongest inhibitory effects on urease, consistent with our findings. This inhibition may be due to the reaction of metal ions with the enzyme’s thiol groups, chelation with the substrate, or interference with the enzyme-substrate complex (Tang et al., 2020). Overall, copper pyrazole effectively inhibits urease activity and nitrification (Li et al., 2022), indicating a promising application for pyrazole-copper in agriculture.

In the present study, the soil mineral N content and microbial traits changed after copper pyrazole application. Our research improves our understanding of the application of copper pyrazole and the microbial mechanisms underlying nitrification and denitrification inhibition by this compound.

### 4.1 Copper pyrazole affects soil mineral N turnover by inhibiting microbial functional genes

The application of nitrification inhibitors is the key agronomic measure that influences soil mineral N content, which is likely regulated by microbial functions (Liu Y. et al., 2014; Woodward et al., 2021). Nitrification is primarily mediated by the AOA-*amoA* and AOB-*amoA* genes, which are largely regulated by ${NH}_{4}^{+}$ -N availability (Cáceres et al., 2018; Chen Z. et al., 2021). Under conditions in which ${NH}_{4}^{+}$ -N levels are not limiting, AOB-*amoA* may play a more dominant role in nitrification than AOA-*amoA* (Prosser and Nicol, 2012; Ouyang et al., 2017). Consistent with these findings, the present study revealed greater AOB-*amoA* gene abundance in the U treatment (Table 1), indicating that the nitrification process was mainly dominated by AOB-*amoA*. Notably, the abundance of the AOB-*amoA* gene was significantly lower in the UC treatment than in the U treatment during incubation (*p* < 0.05, Table 1). This finding suggested that pyrazole may inhibit the first step of nitrification ( ${NH}_{4}^{+}$ -N to hydroxylamine) mainly by inhibiting the activity of AMO, which is encoded by the *amoA* gene, in AOA and AOB (Wrage et al., 2001; Zhang et al., 2013, 2022). Several studies have shown that Cu has direct inhibitory effects on both the abundance of AOA-*amoA* and AOB-*amoA* genes and further inhibits nitrification (Rijk et al., 2023; Upadhyay et al., 2023). As a result, the ${NH}_{4}^{+}$ -N content was significantly greater in the UC treatment than in the U treatment (*p* < 0.05; Figure 1), while the ${NH}_{4}^{+}$ -N content in the SUC treatment was significantly lower than that in the U treatment on the 10th day (*p* < 0.05) and then gradually increased until the end of incubation. This was mainly because of the slow release of N and copper pyrazole from the coated fertilizer, which prolonged the effective treatment time and promoted an increase in the mineral N content (Bröckel and Hahn, 2004; Naz and Sulaiman, 2016). Thus, copper pyrazole retards the transformation of ${NH}_{4}^{+}$ -N to ${\mathrm{NO}}_{3}^{-}$ -N via nitrification by inhibiting AOB-*amoA* gene abundance. Collectively, these findings imply that the chelation complexes formed between pyrazole and copper may significantly inhibit nitrification rates. Therefore, more ${NH}_{4}^{+}$ -N could be preserved in the soil for longer periods of time, enhancing N utilization efficiency. Meanwhile, on the 10th day, the AOA-amoA abundance in the UC treatment was significantly lower than that in the U treatment (*p* < 0.05), possibly due to the addition of copper pyrazole. The study by Shi et al. (2017) also demonstrated that pyrazole derivatives significantly reduced AOA-*amoA* abundance in the spring, consistent with our findings. Whereas on the 60th day, the abundance of the AOA-*amoA* gene in the UC treatment was significantly higher than in the U and SUC treatments (Table 1). This increase might be due to the different rates of urea hydrolysis, as well as the potential role of Cu^2+^ as a cofactor for the AMO enzyme (Glass and Orphan, 2012), which could be released into the soil over time.

The *NirK* and *nosZ* genes have been proven to be involved in nitrite and nitrous oxide reduction, respectively (Bröckel and Hahn, 2004; Naz and Sulaiman, 2016). The *nirK* gene abundance in SUC and UC was lower than that in U on the 60th day due to the inhibitory effect of Cu on denitrification (Liu et al., 2016). Interestingly, the ${\mathrm{NO}}_{3}^{-}$ -N content was significantly greater in the U treatment (*p* < 0.05) than in the SUC and UC treatments on the 60th day, consistent with the differences in *nirK* gene abundance. This is because the presence of copper pyrazole delayed the nitrification process and the associated change from ${NH}_{4}^{+}$ -N to — N, which is consistent with the findings of Li et al. (2022). Moreover, these results were well supported by the significant negative correlation between *nirK* gene abundance and ${NH}_{4}^{+}$ -N content in the correlation analysis (*p* < 0.05, Supplementary Figure S4). Additionally, on the 60th day, the abundance of *nosZ* in the UC and SUC treatments was significantly higher than in the U and CK treatments (*p* < 0.05, Table 1). The findings align with the research by Corrochano-Monsalve et al. (2021), which may be due to Cu^2+^ affecting N_2_O reductase activity (Sullivan et al., 2013). However, the relative abundance of these genes compared to the CK remains within the same order of magnitude, indicating that the denitrification process may not have been significantly affected. Furthermore, the *nirK* gene abundance in SUC was significantly lower than that in UC (*p* < 0.05), probably due to the slow release of copper pyrazole. Thus, copper pyrazole inhibits the denitrification process by inhibiting the abundance of *nirK* genes, thereby prolonging the retention of ${\mathrm{NO}}_{3}^{-}$ -N in soil. This also suggests that the use of coating agents further prolonged the effect of copper pyrazole. In summary, copper pyrazole affects soil mineral N transformation by inhibiting the specific abundance of microbial nitrification and denitrification genes.

### 4.2 The relationship between keystone taxa and mineral N content supported the effect of copper pyrazole on soil N turnover

Soil microbial communities are essential for maintaining soil health and functionality (Fuhrman, 2009), with keystone taxa playing a crucial role in driving the functions of these communities (Ma et al., 2021). Co-occurrence networks are commonly employed in microbial analysis to identify key species that may significantly influence microbial interactions (Duan et al., 2021). Research has demonstrated that keystone taxa-derived specific clusters influence mineral N turnover (Delgado-Baquerizo et al., 2018; Ma et al., 2021; Wang et al., 2022). Therefore, revealing the function of keystone taxa within microbial clusters is vital for clarifying the microbial mechanisms involved in soil N cycling after copper pyrazole application.

In this study, Cluster 4 was significantly correlated (*p* < 0.05) with the soil ${NH}_{4}^{+}$ -N content on the 10th day. Additionally, *Mycobacterium* and *Cronobacter sakazakii* within Cluster 4 were selected for further study. Egamberdiyeva (2007) indicated that *Mycobacteria* promoted the growth of plant roots by facilitating N uptake in nutritionally poor environments and thus accelerated N consumption, which is consistent with our results. Ben Taheur et al. (2016) and Cargnin et al. (2023) demonstrated that *Cronobacter sakazakii* could encode a denitrification gene, which could result in potential N loss. In conclusion, the increased relative abundance of *Mycobacterium* and *Cronobacter sakazakii* may stimulate nitrification by regulating microbial Cluster 4, enhancing the transformation of soil ${NH}_{4}^{+}$ -N to ${\mathrm{NO}}_{3}^{-}$ -N on the 10th day.

In the late stage of incubation, the Cluster 3 community was significantly correlated with the soil ${NH}_{4}^{+}$ -N (*p* < 0.05) and ${\mathrm{NO}}_{3}^{-}$ -N (*p* < 0.001) contents*. Fusariumes* were selected as the keystone taxa within Cluster 3 for further study. Subbarao et al. (2006) demonstrated that *Fusarium* propagules were destroyed more rapidly with ${NH}_{4}^{+}$ -N, particularly following pyrazole application. Moreover, *Fusarium* could markedly contribute to N_2_O emissions (Zheng et al., 2020; Shen et al., 2021). In our study, correlation analysis revealed negative associations between *Fusarium* and both ${NH}_{4}^{+}$ -N and ${\mathrm{NO}}_{3}^{-}$ -N content (*p* < 0.05), which supported these conclusions. Therefore, the Cluster 3 community likely facilitates the denitrification of ${\mathrm{NO}}_{3}^{-}$ -N to form N_2_O.

Notably, the abundance of keystone taxa in Clusters 2 and 4 under copper pyrazole treatment (both UC and SUC) was lower than those in the other treatments on the 10th day (Figure 6). This observation suggested that copper pyrazole may effectively inhibit nitrification by altering the microbial composition and function within these clusters (Gupta et al., 2023), increasing ${NH}_{4}^{+}$ -N accumulation. Additionally, previous studies have shown that nitrapyrin can inhibit keystone taxa (*Nitrobacter* sp.) mediating the second nitrification step, prolonging N fertilizer availability (Woodward et al., 2021). Consistent with these findings, the results of the present study revealed a reduced abundance of Cluster 3 keystone taxa in UC compared to that in the other treatments on the 60th day (Figure 6), suggesting that copper pyrazole may also limit the denitrification of ${\mathrm{NO}}_{3}^{-}$ -N into N_2_O. Further investigations are needed to experimentally verify that the above keystone spices play an important role in the N transformation. Overall, copper pyrazole addition can change the soil N turnover process by altering the relative abundance of keystone taxa. Therefore, the construction of co-occurrence network further supports the role of copper pyrazole in improving the N fertilizer availability from another perspective.

## 5 Conclusion

Overall, copper pyrazole affects soil mineral N transformation by decreasing the abundance of nitrification and denitrification genes and mediating the functions of the soil microbial community. Copper pyrazole exhibited significantly higher inhibition rates on urease compared to other metal-pyrazole coordination compounds, and inhibited the activities of nitrification genes (especially of AOB-*amoA*) and denitrification genes (especially *nirK*), slowing nitrification and denitrification processes, and promoting the accumulation of ${NH}_{4}^{+}$ -N and ${\mathrm{NO}}_{3}^{-}$ -N during incubation.

Moreover, specific taxa play pivotal roles in soil mineral N turnover. In the initial phase, copper pyrazole potentially decreased the relative abundance of *Mycobacterium* and *Cronobacter sakazakii*, suppressing nitrification and retarding ${NH}_{4}^{+}$ -N conversion to ${\mathrm{NO}}_{3}^{-}$ -N. In the late stage, copper pyrazole possibly limited the propagation of *Fusarium*, thereby inhibiting ${\mathrm{NO}}_{3}^{-}$ -N denitrification to N_2_O. This study established the dynamic relationships among copper pyrazole, soil microbial traits and mineral N transformation, providing a scientific and theoretical basis for the application of novel inhibitors.

## Data Availability

The original contributions presented in the study are included in the article/Supplementary material. Raw sequence data obtained in this study have been deposited in the NCBI repository, accession number PRJNA1159739 (https://www.ncbi.nlm.nih.gov/sra/PRJNA1159739).

## Footnotes

^1^ https://www.bioincloud.tech

^2^ http://ieg4.rccc.ou.edu/mena

^3^ http://www.sangerbox.com/tool
