# Supplementary material for: Copper pyrazole addition regulates soil mineral nitrogen turnover by mediating microbial traits
Source: Front Microbiol. 2024 Oct 1;15:1433816. doi: 10.3389/fmicb.2024.1433816 (PMC11473427; doi:10.3389/fmicb.2024.1433816)
Supplement: Supplementary file 1 [file Data_Sheet_1.pdf]

Supplementary Material

Title: Copper pyrazole addition regulates soil mineral nitrogen turnover by mediating microbial traits

Author names: Yuming Wang[1,2,3†], Wenling Zhong[1,2,3†], Xiwen Zhang[1,2,3], Minghui Cao[1,3], Zheng Ni[1,2,3], Mengxia Zhang[1,3,4], Jiangye Li[5], Yan Duan[1,3*], Lifang Wu[1,3*]

[1] The centre for Ion Beam Bioengineering Green Agriculture, Hefei Institutes of Physical Science, Chinese Academy of Sciences, Hefei 230031, Anhui, China
[2] Science Island Branch, Graduate School of USTC, Hefei 230026, China
[3] Zhongke Taihe Experimental Station, Taihe 236626, Anhui, China
[4] School of Life Sciences, Anhui Agricultural University, Hefei 230036, China
[5] Institute of Agricultural Resources and Environment, Jiangsu Academy of Agricultural Sciences, Nanjing 210014, China

* Corresponding author: Yan Duan & Lifang Wu. The centre for Ion Beam Bioengineering Green Agriculture, Hefei Institutes of Physical Science, Chinese Academy of Sciences, Hefei 230031, Anhui, China
E-mail: duanyan@iim.ac.cn & lfwu@ipp.ac.cn, Tel: +86-25-8688-1228, Fax: +86-25-8688-1000.

Supplementary Text S1

Synthesis of the copper pyrazole coordination compound: Pyrazole (2.72 g, 0.04 mol) and $CuCl_2 \cdot 2H_20$ (1.70 g, 0.01 mol) were added to two 25 mL conical flasks, and 5 mL of anhydrous ethanol was subsequently added to the flasks with slight shaking to ensure that the ingredients in the flasks completely dissolved. The above two liquids were combined and stirred for 10 min to make the mixture homogeneous and then left at room temperature for 7 days. A solid product was obtained after the ethanol evaporated completely. Subsequently, the solid product was washed with ethanol and filtered by pumping, which was repeated three times to remove free metal ions and pyrazole to obtain the copper pyrazole coordination compound.

Synthesis of the zinc pyrazole coordination compound， the cobalt pyrazole coordination compound and the cadmium pyrazole coordination compound is the same as that of copper pyrazole coordination compound, only the addition ratios are different, and the ratios are Pyrazole (2.72 g, 0.04 mol) and $ZnCl_2$ (1.36 g, 0.01 mol), Pyrazole (1.36 g, 0.02 mole and $CoCl_2 \cdot 6H_2O$ (2.38 g, 0.01 mol) and Pyrazole (0.68 g, 0.01 mol) and CdI_2 (3.66g, 0.01 mol), respectively.

Supplementary Table S1 Urease inhibition rate of various metal-pyrazole coordination compounds

Treatments	Urease inhibition rate
Cobalt pyrazole coordination compound	4.40±2.13% c
Copper pyrazole coordination compound	99.81±0.10% a
Zinc pyrazole coordination compound	10.73±1.88% b
Cadmium pyrazole coordination compound	9.83±2.00% b

Notes: Values are means ± standard deviation (n = 3) and different letters within the same column denote significant differences ($p < 0.05$) under different treatments.

Supplementary Table S2 Simpson index under different treatments during incubation

Treatments	bacteria		fungi	
	10th day	60th day	10th day	60th day
CK	0.99±0.00046 a	0.99±0.0032 a	0.94±0.024 a	0.96±0.0064 a
U	0.99±0.0015 a	0.99±0.0034 a	0.94±0.013 a	0.95±0.0024 a
UC	0.99±0.0011 b	0.99±0.0034 a	0.91±0.033 a	0.95±0.0068 a
SUC	0.99±0.0011 b	0.99±0.0016 a	0.9±0.036 a	0.96±0.0068 a

Notes: Values are means ± standard deviation (n = 3) and different letters within the same column denote significant differences ($p < 0.05$) under different treatments. CK, Control; U, urea; UC, urea with copper pyrithioxin; SUC, coated urea with copper pyrithioxin.

Supplementary Table S3 The informations of soil microbial keystone taxa

Cultivation time	category	phylum	count	percentage
10th day	bacteria	Proteobacteria	33	49
		Bacteroidetes	10	15
		Acidobacteria	7	10
		Gemmatimonadetes	6	8.8
		Actinobacteria	5	7.4
		Candidatus Saccharibacteria	2	2.9
		Firmicutes	2	2.9
		Chloroflexi	1	1.5
		Thaumarchaeota	1	1.5
		Others	1	1.5
	fungi	Ascomycota	17	71
		Basidiomycota	3	13
		Others	2	8.3
		Chytridiomycota	1	4.2
		Mucoromycota	1	4.2
60th day	bacteria	Proteobacteria	25	58
		Actinobacteria	7	16
		Chloroflexi	5	12
		Acidobacteria	3	7.0
		Bacteroidetes	1	2.3
		Candidatus Saccharibacteria	1	2.3
		Cyanobacteria	1	2.3
	fungi	Ascomycota	4	50
		Others	3	38
		Basidiomycota	1	13

Supplementary Table S4 Soil keystone species taxonomic information

Date	Cluster	ASV	Kingdom	Phylum	Class	Order	Family	Genus	Species
10th day	Cluster2	FASV69	Eukaryota	Ascomycota	Dothideomycetes	Pleosporales	Astrosphaeriellaceae	Pithomyces	Pithomyces chartarum
	Cluster4	BASV129	Bacteria	Actinobacteria	Actinomycetia	Micromonosporales	Micromonosporaceae	Dactylosporangium	-
		BASV159	Bacteria	Actinobacteria	Actinomycetia	Corynebacteriales	Mycobacteriaceae	Mycobacterium	Mycobacterium sp000328565
		BASV197	Bacteria	Proteobacteria	Betaproteobacteria	Burkholderiales	Comamonadaceae	Ramlibacter	Ramlibacter sp013778345
		BASV430	Bacteria	Acidobacteria	Vicinamibacteria	norank	Vicinamibacteraceae	Vicinamibacter	uncultured bacterium
		BASV661	Bacteria	Proteobacteria	Gammaproteobacteria	Enterobacterales	Enterobacteriaceae	Cronobacter	Cronobacter sakazakii
		FASV4	Eukaryota	Ascomycota	Eurotiomycetes	Chaetothyriales	Chaetothyriaceae	Chaetothyrium	Chaetothyrium agathis
		FASV39	Eukaryota	Ascomycota	Eurotiomycetes	Eurotiales	Trichocomaceae	Talaromyces	Talaromyces wortmannii
		FASV59	Eukaryota	Ascomycota	Sordariomycetes	Hypocreales	Niessliaceae	Monocillium	Monocillium mucidum
		FASV65	Eukaryota	Ascomycota	Sordariomycetes	Hypocreales	Stachybotryaceae	Stachybotrys	Stachybotrys limonispora
60th day	Cluster3	FASV15	Eukaryota	Ascomycota	Sordariomycetes	Hypocreales	Nectriaceae	Fusarium	Fusarium cf. solani
		FASV188	Eukaryota	Basidiomycota	Agaricomycetes	Cantharellales	Ceratobasidiaceae	Thanatephorus	Thanatephorus cf. cucumeris F40

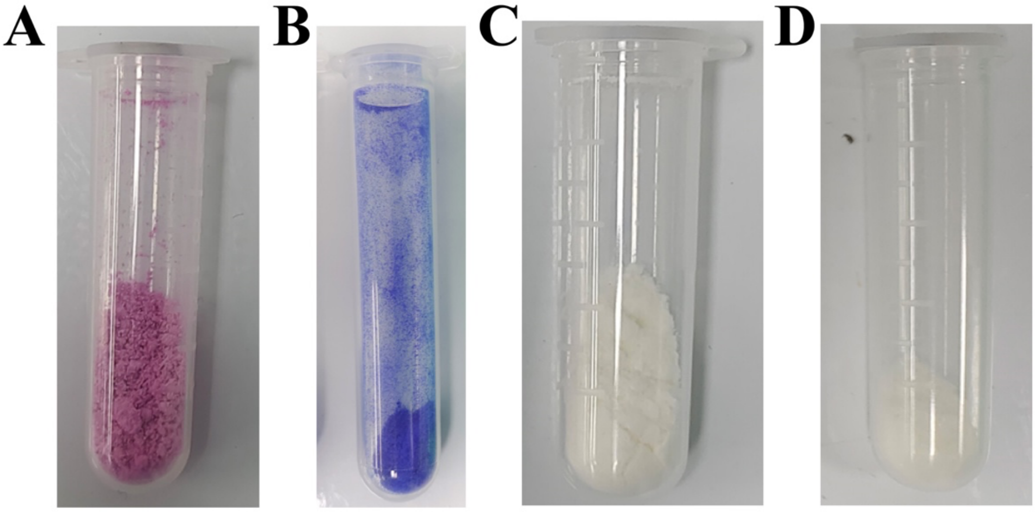

Supplementary Figure S1 The metal-pyrazole coordination compounds, (A) Cobalt pyrazole coordination compound, (B) Copper pyrazole coordination compound, (C) Zinc pyrazole coordination compound, (D) Cadmium pyrazole coordination compound.

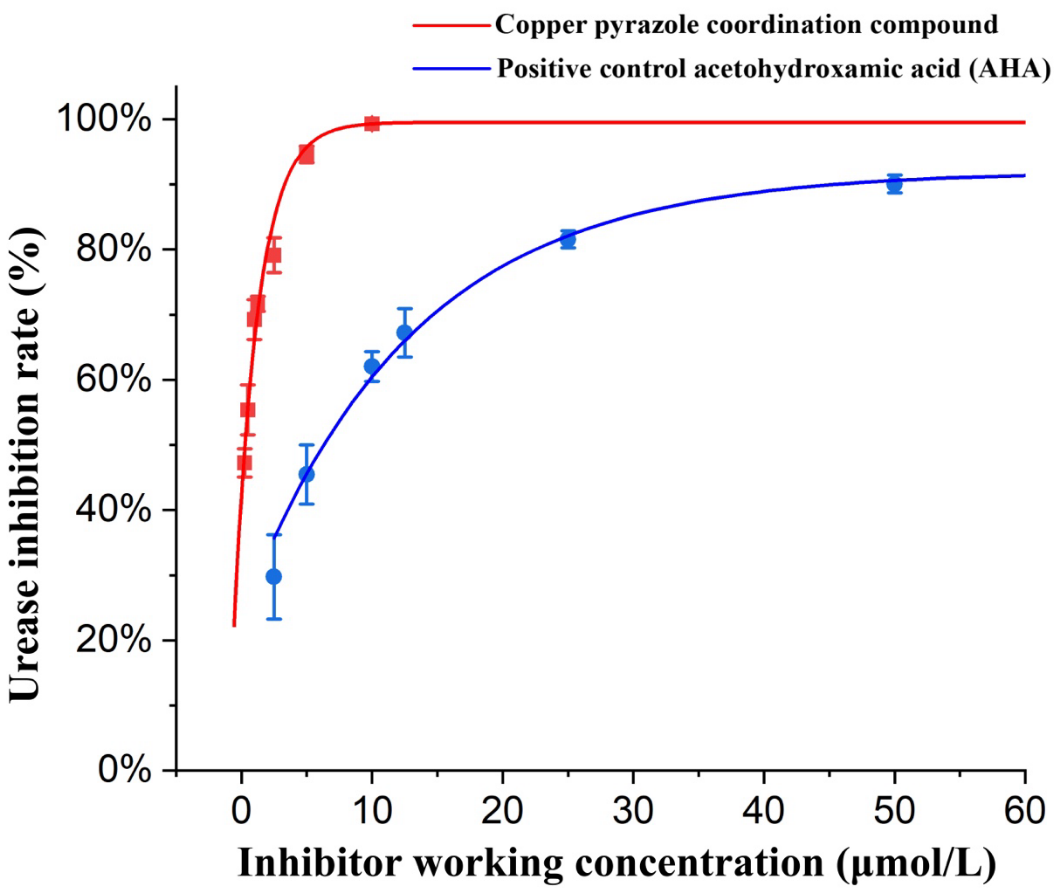

Supplementary Figure S2 Inhibition of urease by copper pyrazole coordination compound and acetohydroxamic acid (AHA) at different concentrations.

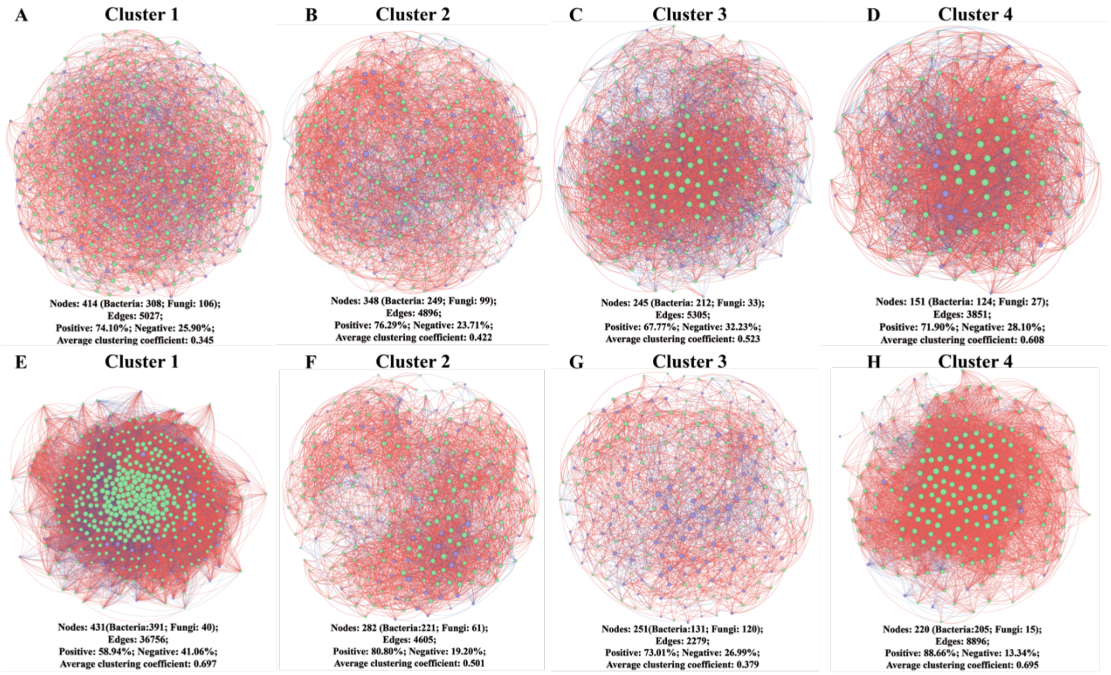

Supplementary Figure S3 Topological characterization of main soil microbial co-occurrence network clusters 1 to 4 at the 10th day (A-D) and the 60th day (E-H) of incubation. The green nodes represent bacteria and the blue nodes represent fungi, the size of each node is proportional to degree (the number of connections); red edges indicate positive, blue edges negative connections.

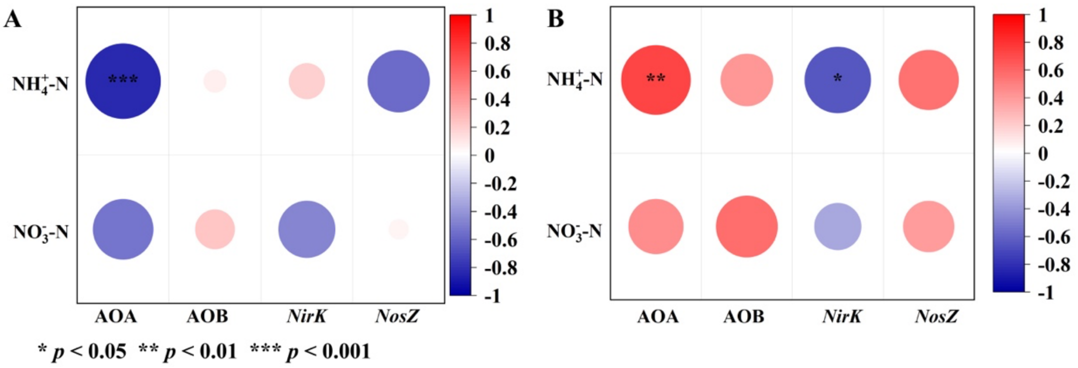

Supplementary Figure S4 Correlation analysis between soil nitrogen and functional genes at the 10th day (A) and the 60th day (B) of incubation. Red indicates a positive correlation, and blue indicates a negative correlation. *, **, and *** indicate significant correlation at $p < 0.05$, $p < 0.01$, and $p < 0.001$.

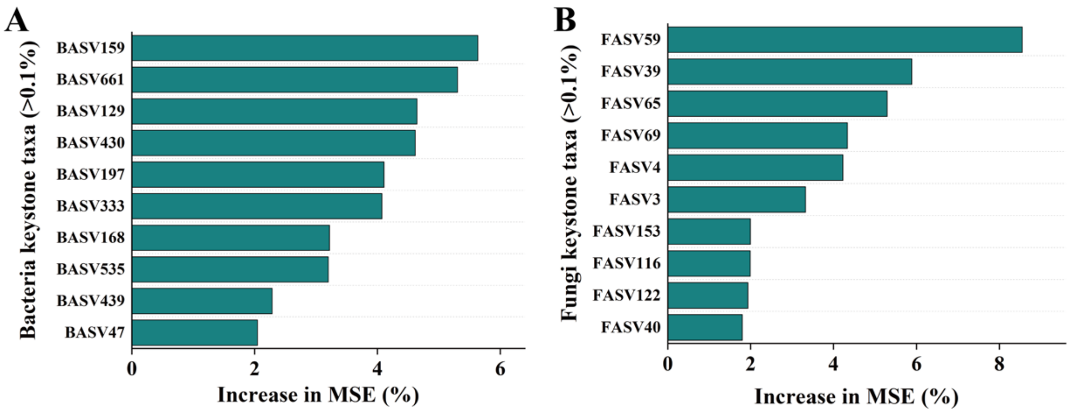

Supplementary Figure S5 Random Forest analysis of bacterial (A) and fungal (B) keystone taxa with the relative abundance > 0.1% in cluster 2 and 4 at the 10th day of incubation

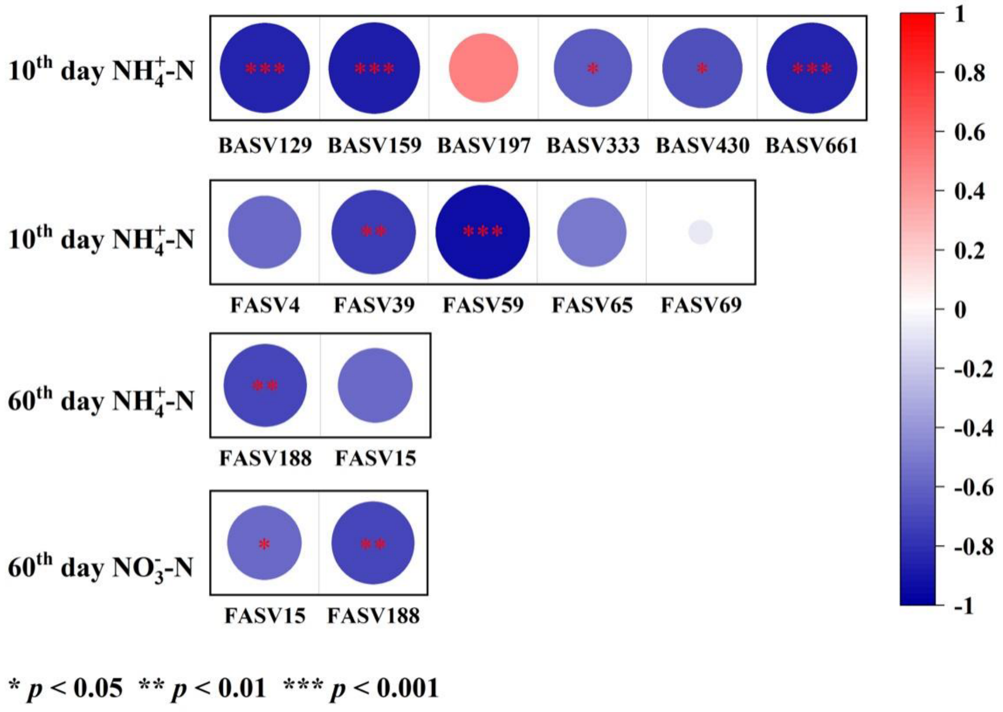

Supplementary Figure S6 Heat map of correlation between microbial keystone species and soil NH_4^+-N and NO_3^--N content at different incubation periods. Red indicates a positive correlation, and blue indicates a negative correlation. *, **, and *** indicate significant correlation at $p < 0.05$, $p < 0.01$, and $p < 0.001$.
